# Countermovement Jump Analysis Using Different Portable Devices: Implications for Field Testing

**DOI:** 10.3390/sports6030091

**Published:** 2018-08-31

**Authors:** Vincenzo Rago, João Brito, Pedro Figueiredo, Thiago Carvalho, Tiago Fernandes, Pedro Fonseca, António Rebelo

**Affiliations:** 1 Center of Research, Education, Innovation and Intervention in Sports, University of Porto, 4099-002 Porto, Portugal; up201005205@fade.up.pt (T.C.); 201301434@fade.up.pt (T.F.); anatal@fade.up.pt (A.R.); 2 Portugal Football School, Portuguese Football Federation, 1495-433 Oeiras, Portugal; joao.brito@fpf.pt (J.B.); pedro.figueiredo@fpf.pt (P.F.); 3 Research Center in Sports Sciences, Health Sciences and Human Development, CIDESD, University Institute of Maia, ISMAI, 4475-690 Maia, Portugal; 4 Porto Biomechanics Laboratory, University of Porto, 4099-002 Porto, Portugal; pedro.labiomep@fade.up.pt

**Keywords:** force platform, motion analysis, neuromuscular assessment, measurements, reliability, validity

## Abstract

The aim of this study was to analyze the concurrent validity, test–retest reliability, and capacity to detect changes of four different portable devices used to measure a wide range of neuromuscular parameters derived from countermovement jump (CMJ). An accelerometric device (Myotest), a jump mat (Ergojump), an optical device (Optojump), and a smartphone app (MyJump) were simultaneously examined for concurrent validity against gold-standard measures (motion-capture system and a force platform). Twenty-two CMJ-derived variables were collected from 15 healthy male subjects (*n* = 60 CMJs). Contraction time (CT) and eccentric duration (EccD) measurements obtained from the Myotest were moderately to largely associated with and not different from force platform measurements (r = 0.31 to 0.64, ES = 0.11 to 0.18) and showed moderate test-retest reliability (intraclass correlation coefficient (ICC) = 0.92 to 0.97, coefficient of variation (CV) = 3.8 to 8.0%). Flight time (FT) and jump height (JH) from Ergojump, Optojump, and MyJump showed moderate to strong associations with gold-standard measurements (r = 0.57 to 0.98) and good test–retest reliability (ICC = 0.54 to 0.97, CV = 1.8 to 4.2). However, all portable devices underestimated JH (ES = 1.25 to 2.75). Independent of the instrument used, the analyzed CMJ variables showed good capacity to detect changes (standard error of measurement (SEM) < smallest worthwhile change (SWC)), with the exception of rate of force and rate of power development parameters, which showed marginal capacity (SEM > SWC). The Myotest is preferable to measure temporal parameters during ground contact, whereas Ergojump, Optojump, and MyJump devices may be preferable to measure FT and JH, with the Optojump being the most accurate.

## 1. Introduction

The countermovement jump (CMJ) is one of the most popular tests to monitor an athlete’s muscle power of the lower extremities. An athlete’s CMJ performance is relevant in a variety of sports and commonly quantified by jump height (JH) or flight time (FT), which have been considered as indicators of vertical jump performance [1,2] and used to measure training adaptations and monitor neuromuscular fatigue [3]. However, CMJ execution is dependent on various time and force components throughout the various phases preceding the flight [4]. Therefore, the use of JH or FT in isolation as a global indicator of muscle power in the lower extremities does not inform about the specific contribution of neuromuscular components during ground contact, consequently masking training-induced adaptations or neuromuscular fatigue. 

CMJ performance variables have been adopted to describe chronic neuromuscular adaptations and acute neuromuscular fatigue in response to exercise, but contradictory results have been observed. Silva et al. [2] examined the correlation between individual match exposure and changes in JH in professional athletes throughout an entire season, reporting no significant training-induced effects. On the other hand, reports in young athletes showed negative associations between various training load parameters (e.g., session rating of perceived exertion exposure time to training) and changes in JH [5]. In addition, JH significantly dropped in response to recreational 5v5 small-sided soccer games [6], whereas CMJ height was not altered following 4v4 and 8v8 small-sided soccer games [7]. Taken together, these findings indicate that JH or FT per se does not elucidate an eventual mask over acute fatigue responses or over chronic adaptations to exercise. In this context, critical information can be directly extracted from the force–time curves during the CMJ, and various parameters (e.g., contraction duration, time to peak force/power) have been recently deemed suitable for neuromuscular fatigue detection. Regarding neuromuscular adaptations, variations in force–time parameters (e.g., flight time to contraction time ratio) were observed in Australian football players over the course of a season, indicating sensitivity to increases with training load over time [8]. Moreover, during a six-week training block with progressive increases in training load, subjective wellness was positively associated with changes in maximum rate of force development, force at zero velocity, and FT (r = 0.28–0.34) in female rugby players [9].

In laboratory conditions, CMJ time and force components may be accurately assessed, providing valid and reliable results using a force platform (FP) [10,11] or video-based motion capture system (MCAP) [1]. For instance, Gathercole et al. [12] examined the reliability and fatigue sensitivity of the CMJ test using an FP, reporting a meaningful reduction in 18 neuromuscular variables obtained from CMJ after a high-intensity fatiguing protocol. These findings suggest that acute fatigue can induce a decrease of peak force and eccentric function and an increase of jump duration [10]. Although FP and MCAP are considered gold standards for CMJ measurement, limited access and associated costs make them unsuitable, especially for field-based testing. Therefore, it would be of interest to understand whether portable equipment frequently used in CMJ testing would allow the assessment of valid and reliable time and force components of CMJ. For example, the Myotest device uses a body accelerometer and has been shown to be a valid and reliable method for the assessment of JH [13,14]. Moreover, the traditional switch mat consists of a digital apparatus connected by a cable to a resistive platform, and can be used on flat surface to test CMJ [15]. Optical systems have been proposed to determine JH by measuring FT [16]. Even more recently, promising results were found from an iPhone app (MyJump), showing excellent validity in relation to FP to measure JH based on the flight-time method [17]. 

No studies have attempted to simultaneously analyze the suitability of a wide range of portable equipment to characterize athletes’ neuromuscular performance. The purpose of this study was to analyze the concurrent validity, test–retest reliability, and capacity to detect changes of four different portable devices used to measure a wide range of CMJ-derived neuromuscular parameters.

## 2. Materials and Methods

### 2.1. Subjects

Fifteen male undergraduate sports sciences students with an average age of 27.0 ± 5.0 years, height of 174.0 ± 9.0 cm, body mass of 72.2 ± 8.28 kg, and training hours of 5.0 ± 2.0 h per week were tested in January 2018. They were regularly involved in various activities such as weight lifting, resistance training, and recreational team sports, and had been injury-free during the last 2 years. All volunteers agreed to provide their maximum effort during all laboratory tests, and they were formally and verbally informed that they were free to withdraw from the study without any consequences. The ethical board of the Faculty of Sports, University of Porto approved and recorded the study under CEFADE.08.2018.

### 2.2. Experimental Design

Four portable devices were examined against the gold standard (FP and MCAP) using 5 CMJs [15] performed by each participant. Each CMJ for each piece of equipment was entered in the database as a single case for intratrial assessment. This methodological approach was used to eliminate the intertrial variability provided by single jump condition performance (i.e., comparisons between independent jump conditions) [13,18]. The independent variable was the device, whereas 22 CMJ-derived parameters, listed in Table 1, were set as the dependent variables. 

Despite the various vertical jump protocols that have been proposed [7,14], CMJ has been considered the most popular and easiest to perform [13]. The subjects received instructions about how to correctly perform CMJ in a separate familiarization session 24 h before data collection. On the data collection day, each subject performed a 5 min cycling warmup on a stationary bike and 2 preliminary CMJs. Then, equipped with an accelerometric system, they were positioned in an upright stance with their feet shoulder-width apart and their toes pointed forward or slightly outward, on the center of the FP, on a switch mat, within optical equipment with light-emitting diodes (LEDs), and in front of a smartphone, simultaneously. The participants executed the jump by flexing their knees to a position they perceived to be comfortable (i.e., preferred starting push-off position) [11,19]. To limit possible variations in posture during the jump that could affect the final assessment, the no-arm swing version was used [13]. At least 30 s recovery was allowed between jump trials.

Eleven kinematic parameters suggested by Gathercole et al. [12] and 11 kinetic parameters previously described elsewhere [4,12,20] were considered (Table 1). All kinetic parameters were scaled to the subject’s body mass.

### 2.3. Procedures

#### 2.3.1. Gold-Standard Measurements

A motion capture system (Qualisys AB, Göteborg, Sweden) operating at a 200 Hz sampling frequency was used. Because CMJ is mainly executed in the sagittal plane, a marker was attached to the left lateral femoral condyle to avoid recording displacement in the frontal plane associated with trunk flexion. This also allowed the mark to be placed as close to the center of mass as possible, enabling tracking along all CMJ phases [19]. This system is capable of ignoring other movement, considering only vertical displacement. The marker displacement, peak, and minimum velocity from the starting position to peak JH were quantified. Second, an extensiometric FP (Bertec FP9060, Bertec, Columbus, KY, USA) embedded in the floor, with a sampling frequency of 2000 Hz, was used. Motion capture and FP data were simultaneously collected using Qualisys Track Manager software (Qualisys AB, Göteborg, Sweden). Force platform variables were computed from the force-velocity, -time, and -acceleration traces using a custom-made MATLAB routine (MATLAB, MathWorks, Natick, MA, USA). According to a specific variable, only 1 criterion (MCAP or FP) was chosen as gold standard.

#### 2.3.2. Accelerometric System

The Myotest Pro (Myotest, Sion, Switzerland) uses a 3-dimensional accelerometer inserted into a small box (dimensions 5.5 × 8.5 × 2.3 cm, mass 50 g) that is secured at waist level with a purpose-built strap (Velcro belt) with an elastic waistband. The Myotest measures acceleration on the vertical axis of the load, on which the accelerometer sensor is fixed, using a sampling rate of 200 Hz. The device was perpendicularly attached to a large (8.5 cm) Velcro elastic belt provided with the device. This was fixed to hip level on the left side of the body, as indicated by the manufacturer, and attached to the left lateral femoral head to reduce the possible accelerations induced by trunk flexion (during the eccentric phase) and extension (i.e., propulsion), thus emphasizing vertical displacement. Accelerometry data were stored during the assessments and subsequently downloaded using the proprietary software (Myotest PRO Software version 1.0, Myotest, Sion, Switzerland), and further neuromuscular variables were computed from raw data of acceleration over time, using a custom-made MATLAB routine (MATLAB, MathWorks, Natick, MA, USA). For the accelerometric system, JH was computed using the flight-time method [10]. 

#### 2.3.3. Switch Mat

The Ergojump mat recorded FT from take-off to landing. The switch mat was placed on top of the floor-embedded force platform and the mat’s weight was removed from the force platform data. This equipment provides JH and FT immediately following the jump. Furthermore, peak power was also calculated using the Sayers equation [21] as follows:

Peak power (W) = 61.9 × JH (cm) + 36.0 × subject’s body mass (kg) + 1.822(1)

#### 2.3.4. Optical Equipment with Light-Emitting Diodes 

The Optojump system (Optojump, Microgate, Bolzano, Italy) consists of 2 bars (transmitting and receiving bars, 1 m apart) equipped with 33 optical LEDs fitted in the transmitting bar that continuously communicate with the corresponding set in the receiving bar. The LEDs are positioned 0.3 cm from ground level, on the Ergojump mat, and at 3.125 cm intervals. Any break of the beam switched on and off automatically activates a digital chronometer used to calculate FT and JH [10].

#### 2.3.5. Smartphone App

The MyJump app was installed on an iPhone 6, which includes a 120 Hz high-speed camera, at a quality of 720 p. MyJump was designed to calculate the time (in ms) between 2 frames selected by the user and subsequently JH using the following equation:

JH = FT^2^ × 1.22625 (2)

A researcher lay prone on the ground with the iPhone 6 facing the participant (in the frontal plane) ~1.5 m from the force platform at floor level, and zoomed in on the feet of the participant. The MyJump app provides JH, FT, and peak velocity. Peak power was also computed as previously described.

### 2.4. Statistical Analysis

In accordance with the outlier removal procedure suggested by Gathercole et al. [12], we selected the 4 out of 5 most consistent CMJ repetitions (differing least from the mean) based on mean eccentric and concentric power over time. Therefore, only 4 CMJs collected were considered for analysis, resulting in 60 cases across the 75 jumps performed. All variables were normally distributed, and data are reported as mean ± standard deviation (SD).

Correlations between the gold standard and portable devices were quantified using the following criteria: ≤0.1 (trivial), 0.1–0.3 (small), 0.3–0.5 (moderate), 0.5–0.7 (large), 0.7–0.9 (very large), and ≥0.9 (almost perfect). Substantial over- and underestimations were analyzed using paired sample *t*-test. Differences were interpreted using effect size (ES) according to Hopkins [22] as trivial (<0.2), small (0.2–0.6), moderate (0.6–1.2), large (1.2–2.0), very large (2.0–4.0), and huge (>4.0). When 90% confidence intervals (CIs) overlapped positive and negative values, the effect was deemed as unclear. Otherwise, the magnitude is reported as the observed value. A large correlation (r ≥ 0.90) and a trivial difference (ES < 0.2) indicated sufficient validity. Test–retest reliability was assessed using intraclass correlation coefficient (ICC) (absolute reliability [1,2], a 2-way random effects model with single measure) and coefficients of variation (CV; relative reliability). For ICC, we used criteria suggested by Portney and Watkins [23] to evaluate reliability as follows: ≥0.75 (good), 0.50–0.74 (moderate), 0.26–0.50 (fair), and ≤0.25 (poor). CV was calculated by dividing the within-subjects SD by the mean and then multiplying by 100, qualitatively classified as good (<5%), moderate (5–10%), and poor (>10%). ICC ≥ 0.75 and CV ≤ 10% were defined as sufficient reliability. Standard error of measurement (SEM) was calculated by dividing the SD of the difference score between test and retest by $\sqrt{\mathrm{ICC}}$ [24]. The smallest worthwhile change (SWC) was determined to establish the usefulness of the test by multiplying the between-subject standard deviation by 0.2. If SEM is smaller than SWC, the ability to detect a change is “good”; if SEM equals SWC, then the test is “satisfactory”; but if SEM is greater than SWC, then the test is rated as “marginal” [25]. The minimal detectable change (MDC_95_) was calculated as MDC_95_ = SEM × $\sqrt{2}$ × 1.96 [25].

## 3. Results

**Figure 1:**
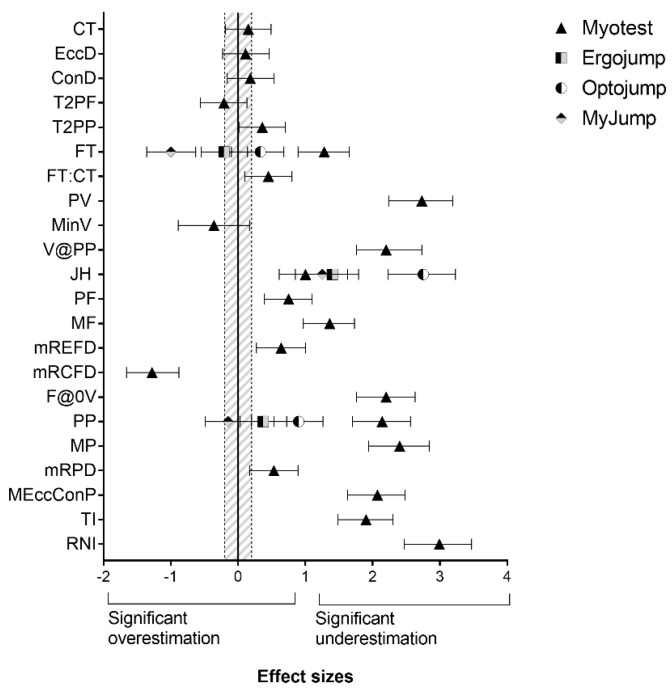
Differences between gold-standard measurements and four portable devices in 22 neuromuscular parameters. Shaded area indicates trivial differences (ES < 0.2). ConD, concentric duration; CT, total contraction time; EccD, Eeccentric duration; ES, effect size; F@0V, force at zero velocity; FT, flight time; FT:CT, flight time to contraction time ratio; ICC, intraclass correlation coefficient; MF, mean force; MP, mean power; MEccConP, mean eccentric and concentric power over time; MinV, minimum velocity; mRCFD, maximum rate of concentric force development; mREFD, maximum rate of eccentric force development; mRPD, maximum rate of power development; PF, peak force; PP, peak power; PV, peak velocity; RNI, relative net impulse; T2PF, time to peak force; T2PP, time to peak power; TI, total impulse; V@PP, velocity at peak power. Gold standard is a motion capture system for PV, MinV, and JH, and force platform for all remaining variables.

**Figure 2:**
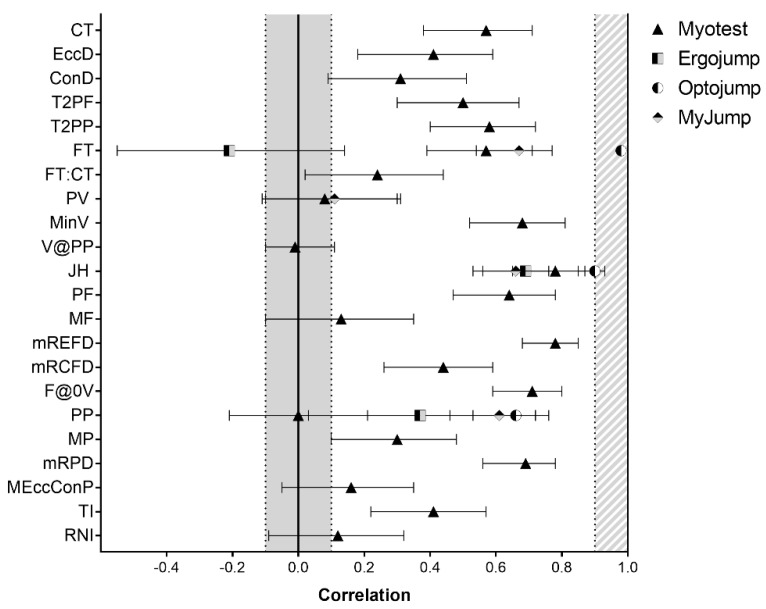
Correlations between gold standard measurements and four portable devices in 22 neuromuscular parameters. Filled gray area indicates no significant correlation (r < 0.1). Shaded space indicates almost perfect correlation (r ≥ 0.9). ConD, concentric duration; CT, total contraction time; EccD, eccentric duration; ES, effect size; F@0V, force at zero velocity; FT, flight time; FT:CT, flight time to contraction time ratio; ICC, intraclass correlation coefficient; MF, mean force; MP, mean power; MEccConP, mean eccentric and concentric power over time; MinV, minimum velocity; mRCFD, maximum rate of concentric force development; mREFD, maximum rate of eccentric force development; mRPD, maximum rate of power development; PF, peak force; PP, peak power; PV, peak velocity; RNI, relative net impulse; T2PF, time to peak force; T2PP, time to peak power; TI, total impulse; V@PP, velocity at peak power. Gold standard is a motion capture system for PV, MinV, and JH, and force platform for all remaining variables.

Descriptive values are reported in Table 2. Differences and correlations between gold standard and portable devices are reported in Figure 1 and Figure 2, respectively. 

CT, EccD, concentric duration (ConD), and peak force (PF) obtained from the Myotest were moderately to largely correlated and not significantly different from FP measurements (r = 0.31–0.64, ES = 0.11–0.18). In addition, FT, JH, PF, maximum rate of eccentric force (mREFD), maximum rate of concentric force development (mRCFD), force at zero velocity (F@0V), maximum rate of power development (mRPD), and time to peak power (TI) measurements were positively correlated with but underestimated FP measurements (r = 0.44–0.71, ES = 0.53–2.20). The remaining variables were not valid when compared to gold-standard measurements. FT obtained from Ergojump was positively correlated with and not different from FP (r = 0.61 [0.09; 0.32], ES = 0.21 [−0.55; 0.14]). In contrast, JH was strongly associated with but substantially different from FP (r = 0.69; ES = 1.40 [1.00; 1.79]). FT obtained from Optojump was valid in relation to FP (r = 0.98 [0.97; 0.99], ES = 0.33 [−0.01; 0.68]). Despite JH from Optojump being almost perfectly correlated with these devices, they were significantly different (r = 0.90; ES = 2.75 [2.23; 3.23]). FT and JH obtained from MyJump were strongly associated with FP and MCAP (r = 0.67 [0.54; 0.67] and 0.66 [0.53; 0.76], respectively). However, FT overestimated FP, and JH underestimated MCAP measurements (ES = −0.1.00 [−1.36; −0.63] and 1.25 [0.85; 1.63], respectively). Estimated PP obtained from MyJump was largely associated with and not different from PP from MCAP (r = 0.61 [0.46; 0.72]; ES = −0.15 [−0.49; −0.20]). A huge underestimation was found for MyJump-derived PV compared to MCAP (ES = 9.65 [8.05; 11.06]). Estimated PP assessed via all devices was not valid in relation to FP measurements.

A detailed description of test–retest reliability is reported in Table 3. FT and JH were reliable for all portable devices (ICC = 0.54–0.97, CV = 1.8–4.2%). CT, EccD, time to peak force (T2PF), time to peak power (T2PP), flight time to contraction time ratio (FT:CT), PF, MF, F@0V, and mean eccentric and concentric power over time (MEccConP) obtained from Myotest showed good absolute and relative reliability (ICC = 0.89–0.97, CV = 3.8–9.5%). PV obtained from MyJump also showed good test–retest reliability (ICC [95% CI] = 0.97 [0.91; 0.99]; CV = 3.9%). With the exception of Myotest, PP was reliable for all portable devices (ICC = 0.84–0.91, CV = 3.3–4.5%). In all devices, all variables showed good capacity to detect changes (SEM < SWC), except for mREFD, mRCFD, and mRPD (SEM > SWC) (Table 4).

## 4. Discussion

To best of our knowledge, this is the first study assessing the validity and reliability of different portable devices to measure CMJ-derived parameters. Different instruments may be used in the field according to the targeted neuromuscular parameter. Specifically, Myotest is valuable for measuring CMJ-derived temporal parameters during ground contact (e.g., CT and EccD), whereas Ergojump, Optojump, and MyJump may be preferable for measuring FT and JH. On the other hand, FT obtained from Optojump was the only variable that was either valid (in relation to gold-standard devices) and reliable (test–retest) to measure CMJ performance. FT should be used as the criterion measure of jump performance instead of JH, since it is directly measured. Indeed, JH is indirectly obtained from an equation that uses the flight time squared [10], which means that the measurement error increases with longer flight times. It should also be noted that the equipment tested for reliability and validity showed good sensitivity to detecting changes in all variables measured, with the exception of mREFD, mRCFD, and mRPD.

In this study, we used a within-subject design to avoid interjump variability. It has been suggested that method agreement–type studies should involve at least 40 participants for adequate statistical accuracy [25]. Despite our smaller number of participants, repeated measurements on individual subjects (four jumps per subject) resulted in 60 cases, increasing statistical accuracy [26].

Despite the wide use of portable devices, only a few independent research studies have addressed their reliability and validity [13,14,17,18,27]. One interesting finding of this research is the validity and reliability of some CMJ-derived parameters (CT, EccD, ConD, PF) obtained from Myotest, possibly of interest for fatigue-monitoring purposes. Gathercole et al. [12] reported small changes in EccD (increase) and PF (decrease) immediately after exercise (three yo-yo intermittent endurance tests separated by 5 min active recovery), possibly indicating good capacity of these parameters to detect neuromuscular fatigue. Similarly, our study showed that Myotest-derived CT, EccD, ConD, and PF can be used to track changes in neuromuscular function throughout recovery. In addition, reports in professional soccer players have shown that fluctuations in high-intensity running (>14.4 km∙h^−1^) on the previous day were associated with CMJ-derived JH fluctuations [3]. However, this correlation was small (r = 0.23), and therefore we speculate that other neuromuscular parameters may be more sensitive for detecting neuromuscular fatigue induced by training load, rather than JH. Therefore, future studies should employ Myotest in the field on a daily basis and analyze with CMJ-derived variables to investigate fatigue response to training.

It should also be noted that FT, JH, F@0V, FTTPF, TTPP, and FT:CT showed good test–retest reliability in all portable devices, but significant differences from FP measurements. This possibly indicates that these parameters are less accurate for assessing training adaptations and monitoring fatigue. Our results on FT and JH as CMJ performance indicators corroborates results of previous attempts investigating the concurrent validity of Myotest in relation to FP [13] or even to Optojump when assumed as gold standard [14]. Specifically, our study showed that JH evaluated in Myotest was overestimated in Optojump by ~12 cm. This was also observed in previous efforts to concurrently validate assessment of Myotest, employing indirect estimations of JH (calculated from vertical take-off velocity) [14]. However, when employing the FT equation instead of taking CMJ data from the Myotest display (based on take-off velocity), JH can be more accurately estimated [10]_ENREF_9. Indeed, our results show that FT measured by Myotest is neither valid nor reliable. This could partially be attributed to the different methodologies adopted. In the present study, different methods were adopted using direct measurement of JH and FT (by MCAP), possibly obtaining more accurate results than when using a portable device as the gold standard (e.g., Optojump) [14].

Although the results obtained from Ergojump showed a lack of concurrent validity in relation to gold-standard devices (r = 0.61; [<0.90]) it accomplished the reliability assumptions (ICC = 0.54 [>0.50]; CV = 8.2 [<10%]). This is likely because the switch mat directly computes FT using the time taken from take-off to landing. On the other hand, the system indirectly calculates JH based on flight time, possibly explaining observed differences (<8 cm) compared to the MCAP. Future studies may implement a more rigorous protocol with more jump trials, which could contribute to increased reliability.

Sharing the same method of calculation, JH outcomes from Optojump and Ergojump are similar, except for validity (r = 0.98 and 0.61, respectively). Indeed, FT obtained from Optojump was almost perfectly correlated, reliable, and not different compared to FP. This is in accordance with Castagna et al. [13], who observed a nearly perfect correlation (r = 0.99) and no significant differences between FT measured in Optojump and FP. Moreover, Glatthorn et al. [16] reported a correlation coefficient of 0.99 between Optojump and FP for JH values, even if a systematic bias of ~1 cm was observed. In addition, previous reports showed excellent test–retest reliability (ICC = 0.98, CV = 2.7%) of JH from Optojump and low random error between repetitions (~2.8 cm) [16].

In the present study, despite large association and good reliability, FT and JH obtained from MyJump were significantly different from gold-standard devices. JH findings are partially in agreement with previous reports showing either significant associations (r = 0.96–0.99) or no differences between MyJump and FP [17,28]. In this study, differences in JH between MyJump and gold-standard devices could be attributed to the fact that the take-off and landing frame selection in MyJump was performed manually, which could increase the measurement error. Fortunately, the continued progress of technology indicates that in the near future smartphones will include cameras with higher recording frequencies, which may reduce the measurement error of MyJump.

The rate of force and rate of power development indicate how rapidly force or power changes (i.e., rate of change) from the beginning to the end of the CMJ. Reliability statistics showed that mRCFD, mRCED, and mRPD have larger variability than the other variables. These findings are supported by previous research investigating the test–retest reliability of mRFD (CV = 16–35%) [20]. This inherent variability may be explained by dynamic system theory, which suggests that movement coordination is adjusted for perturbation to obtain a consistent performance outcome, and therefore both the rate of force and the rate of power development during CMJ may be more reflective of coordination adjustment than the characteristics of the lower extremities [20].

As a main limitation, the subjects differed in sports activities (e.g., weight lifting, resistance training, and recreational team sports), possibly affecting our results. Therefore, future studies should explore the reliability of these parameters in professional athletes.

Athletes are subject to frequent alterations to their training schedule, according to coaches’ decisions and the competitive calendar. Therefore, another important aspect of field testing is the flexibility allowed by the coach to change assessment scheduling, without any commitment to external laboratories. We used the standardized CMJ version (hands remained in contact with the spine of the pelvis) to analyze test–retest reliability. Future studies may use the arm-swing CMJ version to verify whether the jump technique affects the reliability of variables, as well as further novel systems (e.g., Xbox Kinect).

## 5. Conclusions

This study compares a wide range of CMJ-derived variables over six different devices, aiming to analyze the most convenient tool to be employed for on-field neuromuscular testing. Although the portable devices showed different levels of accuracy to measure CMJ according to the specific neuromuscular parameters analyzed, Myotest should be preferred to measure CMJ-derived temporal parameters during ground contact (e.g., CT and EccD). Ergojump, Optojump, and MyJump quantify the time taken from jump take-off to landing and may be adopted to measure FT or JH. Optojump seems to show the most accurate CMJ performance measurement across these devices. Finally, with the exception of rate of force and rate of power development, all parameters analyzed in the current study showed good capacity to detect changes irrespective of the equipment used. The application of portable devices could be of interest for coaches and practitioners to schedule and change testing or training sessions according to the team’s or player’s calendar, without any commitment to an external laboratory.

## Figures and Tables

**Table 1:** Definitions of neuromuscular parameters adopted.

Variables	Abbreviation	Unit of Measure	Definition
*Kinematic parameters*	-	-	-
Total contraction time	CT	s	Total duration from jump initiation to take-off
Eccentric duration	EccD	s	Time period of the eccentric phase
Concentric duration	ConD	s	Time period of the concentric phase
Time to peak force	T2PF	s	Time period between jump start and peak force
Time to peak power	T2PP	s	Time period between jump start and peak power
Flight time	FT	s	Time period of zero force, corresponding to noncontact with the floor
Flight time to contraction time ratio	FT:CT	%	Ratio between flight time and contraction time
Peak velocity	PV	m∙s^−1^	Highest jump velocity during the concentric phase
Minimum velocity	MinV	m∙s^−1^	Lowest jump velocity during the eccentric phase
Velocity at peak power	V@PP	m∙s^−1^	Velocity recorded at time point where peak power occurred
Jump height	JH	m	Highest height achieved during the jump
*Kinetic parameters*	-	-	-
Peak force	PF	N∙kg^−1^	Maximum force achieved during the concentric phase
Mean force	MF	-	Average force of the concentric phase
Maximum rate of eccentric force development	mREFD	N∙s^−1^∙kg^−1^	Maximum force increase within a 30 ms window during the eccentric phase
Maximum rate of concentric force development	mRCFD	N∙s^−1^∙kg^−1^	Maximum force increase within a 30 ms window during the concentric phase.
Force at zero velocity	F@0V	N∙kg^−1^	Force exerted at the end of the countermovement where the jump transitions from eccentric to concentric movement
Peak power	PP	W∙kg^−1^	Higher power value achieved during the jump
Mean power	MP	W∙kg^−1^	Average power value obtained during the concentric phase
Maximum rate of power development	mRPD	W∙s^−1^∙kg^−1^	Largest power increase within a 30 ms window during the concentric phase
Mean eccentric and concentric power over time	MEccConP	W∙kg^−1^∙s^−1^	Power produced during both eccentric (converted to positive values) and concentric phases, divided by contraction duration
Total impulse	TI	N∙s^−1^	Force exerted concentrically multiplied by the time taken concentrically
Relative net impulse	RNI	N∙s∙kg^−1^	Impulse between the beginning of the concentric phase and the moment force reached weight level, minus the impulse equivalent to that of the propulsion-deceleration phase

**Table 2:** Descriptive values of countermovement jump (CMJ) kinematic variables obtained with different devices. Values are mean ± standard deviation (SD).

Variable	MCAP	Force Platform	Myotest	Ergojump	Optojump	MyJump
*Kinematic parameters*						
CT (s)	-	0.77 ± 0.16	0.76 ± 0.09	-	-	-
EccD (s)	-	0.50 ± 0.15	0.50 ± 0.08	-	-	-
ConD (s)	-	0.26 ± 0.02	0.26 ± 0.04	-	-	-
T2PF (s)	-	0.55 ± 0.17	0.60 ± 0.10	-	-	-
T2PP (s)	-	0.71 ± 0.16	0.67 ± 0.09	-	-	-
FT (s)	-	0.54 ± 0.03	0.49 ± 0.04	0.55 ± 0.06	0.53 ± 0.03	0.57 ± 0.03
FT:CT (%)	-	0.72 ± 0.16	0.66 ± 0.10	-	-	-
PV (m∙s^−1^)	3.04 ± 0.23	-	2.08 ± 0.39	-	-	1.42 ± 0.23
MinV (m∙s^−1^)	−1.25 ± 0.16	-	−1.08 ± 0.26	-	-	-
V@PP (m∙s^−1^)	-	2.47 ± 0.15	1.89 ± 0.34	-	-	-
JH (m)	0.46 ± 0.04	-	0.42 ± 0.04	0.38 ± 0.07	0.35 ± 0.04	0.41 ± 0.04
*Kinetic parameters*						
PF (N∙kg^−1^)	-	23.88 ± 1.99	22.23 ± 2.37	-	-	-
MF (N∙kg^−1^)	-	13.39 ± 1.24	11.96 ± 0.82	-	-	-
mREFD (N∙s^−1^∙kg^−1^)	-	136.88 ± 69.88	101.27 ± 36.22	-	-	-
mRCFD (N∙s^−1^∙kg^−1^)	-	29.37 ± 19.93	57.44 ± 23.82	-	-	-
F@0V (N∙kg^−1^)	-	23.16 ± 2.31	18.77 ± 3.30	-	-	-
PP (W∙kg^−1^)	-	36.01 ± 8.80	47.84 ± 10.17	46.18 ± 5.38	51.47 ± 3.80	-
MP (W∙kg^−1^)	-	28.66 ± 2.51	18.89 ± 5.17	-	-	-
mRPD (W∙s^−1^∙kg^−1^)	-	345.49 ± 81.79	301.08 ± 84.41	-	-	-
MEccConP (W∙kg^−1^∙s^−1^)	-	13.63 ± 2.29	9.14 ± 2.04	-	-	-
TI (N∙s^−1^)	-	197.00 ± 29.77	121.74 ± 47.34	-	-	-
RNI (N∙s^−1^∙kg^−1^)	-	2.61 ± 0.16	1.70 ± 0.40	-	-	-

MCAP, motion capture system; ConD, concentric duration; CT, total contraction time; EccD, eccentric duration; F@0V, force at zero velocity; FT, flight time; FT:CT, flight time to contraction time ratio; MP, mean power; MEccConP, mean eccentric and concentric power over time; MinV, minimum velocity; mRCFD, maximum rate of concentric force development; mRCED, maximum rate of eccentric force development; mRPD, maximum rate of power development; PF, peak force; PP, peak power; PV, peak velocity; RNI, relative net impulse; T2PF, time to peak force; T2PP, time to peak power; TI, total impulse; V@PP, velocity at peak power.

**Table 3:** Test–retest reliability of CMJ kinematic variables obtained with two gold-standard pieces of equipment and four portable devices.

Variable	Reliability Indicators	MCAP	Force Platform	Myotest	Ergojump	Optojump	MyJump
CT	ICC (95% CI)	-	0.84 (0.58; 0.95)	0.93 (0.83; 0.98)	-	-	-
	CV (%)	-	120.1 (7.7; 16.5)	6.8 (4.9; 8.7)	-	-	-
EccD	ICC (95% CI)	-	0.81 (0.51; 0.94)	0.95 (0.87; 0.98)	-	-	-
	CV (%)	-	17.5 (10.1; 24.9)	8.0 (5.7; 10.3)	-	-	-
ConD	ICC (95% CI)	-	0.89 (0.71; 0.96)	0.72 (0.27; 0.92)	-	-	-
	CV (%)	-	6.8 (5.2; 8.3)	12.0 (7.1; 16.9)	-	-	-
T2PF	ICC (95% CI)	-	0.80 (0.48; 0.94)	0.93 (0.82; 0.98)	-	-	-
	CV (%)	-	21.1 (14.4; 27.7)	7.9 (5.8; 10.1)	-	-	-
T2PP	ICC (95% CI)	-	0.82 (0.52; 0.95)	0.93 (0.83; 0.98)	-	-	-
	CV (%)	-	13.3 (8.6; 18.1)	7.0 (5.1; 8.9)	-	-	-
FT	ICC (95% CI)	-	0.97 (0.92; 0.99)	0.96 (0.91; 0.99)	0.54 (−0.34; 0.89)	0.96 (0.91; 0.99)	0.80 (0.37; 0.96)
	CV (%)	-	1.8 (1.3; 2.2)	3.8 (0.4; 7.2)	8.2 (0.8; 15.7)	1.8 (1.4; 2.3)	3.0 (2.1; 3.8)
FT:CT	ICC (95% CI)	-	0.73 (0.30; 0.92)	0.92 (0.78; 0.98)	-	-	-
	CV (%)	-	13.2 (8.0; 18.3)	8.4 (3.4; 13.4)	-	-	-
PV	ICC (95% CI)	0.91 (0.78; 0.97)	-	0.78 (0.44; 0.94)	-	-	0.74 (0.17; 0.95)
	CV (%)	5.2 (3.2; 7.1)	-	11.6 (7.6; 15.3)	-	-	3.3 (2.2; 4.4)
MinV	ICC (95% CI)	0.79 (−39; 0.95)	-	0.50 (−0.30; 0.86)	-	-	-
	CV (%)	−6.8 (−8.0; −5.6)	-	−18.0 (−24.6; −12.3)	-	-	-
V@PP	ICC (95% CI)	-	0.97 (0.92; 0.99)	0.79 (0.46; 0.94)	-	-	-
	CV (%)	-	2.1 (1.54; 2.6)	11.1 (7.7; 14.5)	-	-	-
JH	ICC (95% CI)	0.96 (0.90; 0.99)	-	0.97 (0.92; 0.99)	0.93 (0.81; 0.98)	0.87 (0.62; 0.97)	0.97 (0.91; 0.99)
	CV (%)	3.3 (2.2; 4.4)	-	4.2 (3.2; 5.3)	3.2 (2.3; 4.1)	4.2 (3.3; 5.1)	3.9 (3.0; 4.9)
PF	ICC (95% CI)	-	0.83 (0.57; 0.95)	0.91 (0.77; 0.97)	-	-	-
	CV (%)	-	3.4 (2.4; 4.5)	6.4 (4.4; 8.3)	-	-	-
MF	ICC (95% CI)	-	0.89 (−0.73; 0.96)	0.89 (0.70; 0.96)	-	-	-
	CV (%)	-	5.2 (1.9; 8.5)	4.2 (2.5; 5.9)	-	-	-
mREFD	ICC (95% CI)	-	0.89 (0.71; 0.97)	0.76 (0.37; 0.93)	-	-	-
	CV (%)	-	17.1 (10.8; 23.4)	21.1 (15.3; 26.8)	-	-	-
mRCFD	ICC (95% CI)	-	0.84 (0.62; 0.95)	0.28 (−0.29; 0.86)	-	-	-
	CV (%)	-	46.4 (30.4; 62.4)	30.9 (20.9; 40.9)	-	-	-
	*F@0V*	-	-	-	-	-	-
F@0V	ICC (95% CI)	-	0.90 (0.74; 0.97)	0.91 (0.77; 0.97)	-	-	-
	CV (%)	-	4.3 (2.7; 5.9)	8.9 (5.6; 12.2)	-	-	-
PP	ICC (95% CI)	-	0.95 (0.87; 0.98)	0.81 (0.52; 0.94)	0.91 (0.79; 0.97)	0.86 (0.63; 0.96)	0.84 (0.48; 0.96)
	CV (%)	-	3.4 (2.1; 4.7)	15.8 (11.3; 20.4)	3.3 (2.3; 4.3)	4.5 (1.0; 8.1)	4.0 (3.0; 5.0)
MP	ICC (95% CI)	-	0.91 (0.77; 0.97)	0.84 (0.59; 0.95)	-	-	-
	CV (%)	-	3.8 (2.7; 5.0)	14.8 (10.2; 19.3)	-	-	-
	*mRPD*	-	-	-	-	-	-
mRPD	ICC (95% CI)	-	0.75 (0.34; 0.93)	0.84 (0.58; 0.95)	-	-	-
	CV (%)	-	10.1 (8.0; 12.3)	16.0 (10.6; 21.3)	-	-	-
MEccCONP	ICC (95% CI)	-	0.87 (0.65; 0.96)	0.93 (0.81; 0.98)	-	-	-
	CV (%)	-	9.0 (5.9; 12.1)	9.5 (5.8; 13.2)	-	-	-
TI	ICC (95% CI)	-	0.79 (0.44; 0.94)	0.95 (0.87; 0.98)	-	-	-
	CV (%)	-	5.6 (1.3; 9.9)	24.4 (10.8; 37.9)	-	-	-
RNI	ICC (95% CI)	-	0.97 (0.92; 0.99)	0.86 (0.63; 0.96)	-	-	-
	CV (%)	-	1.7 (1.1; 2.2)	15.9 (10.1; 21.8)	-	-	-

MCAP, motion capture system; ConD, concentric duration; CT, total contraction time; EccD, eccentric duration; F@0V, force at zero velocity; FT, flight time; FT:CT, flight time to contraction time ratio; MP, mean power; MEccConP, mean eccentric and concentric power over time; MinV, minimum velocity; mRCFD, maximum rate of concentric force development; mRCED, maximum rate of eccentric force development; mRPD, maximum rate of power development; PF, peak force; PP, peak power; PV, peak velocity; RNI, relative net impulse; T2PF, time to peak force; T2PP, time to peak power; TI, total impulse; V@PP, velocity at peak power. Bold indicates reliable measurements (ICC > 0.75, CV < 10%).

**Table 4:** Standard error of measurement, smallest worthwhile change, and minimum detectable change of jump variables obtained with different devices.

Variable	MCAP			Force Platform			Myotest			Ergojump			Optojump			MyJump		
	SEM	SWC	MDC	SEM	SWC	MDC	SEM	SWC	MDC	SEM	SWC	MDC	SEM	SWC	MDC	SEM	SWC	MDC
*Kinematic parameters*																		
CT (s)	-	-	-	0.02	0.03	0.05	0.012	0.018	0.033	-	-	-	-	-	-	-	-	-
EccD (s)	-	-	-	0.01	0.03	0.05	0.010	0.016	0.029	-	-	-	-	-	-	-	-	-
ConD (s)	-	-	-	0.003	0.006	0.01	0.005	0.008	0.015	-	-	-	-	-	-	-	-	-
T2PF (s)	-	-	-	0.02	0.03	0.06	0.013	0.021	0.038	-	-	-	-	-	-	-	-	-
T2PP (s)	-	-	-	0.02	0.03	0.06	0.011	0.018	0.033	-	-	-	-	-	-	-	-	-
FT (s)	-	-	-	0.004	0.006	0.01	0.005	0.008	0.015	0.008	0.013	0.024	0.004	0.006	0.012	0.003	0.006	0.010
FT:CT (%)	-	-	-	0.02	0.03	0.05	0.013	0.021	0.038	-		-	-	-	-	-	-	-
PV (m∙s^−1^)	0.04	0.06	0.11	-	-	-	0.050	0.078	0.14	-		-	-	-	-	-	-	-
MinV (m∙s^−1^)	0.02	0.03	0.05	-	-	-	0.032	0.050	0.090	-		-	-	-	-	-	-	-
V@PP (m∙s^−1^)	-	-	-	0.02	0.03	0.05	0.04	0.06	0.056	-		-	-	-	-	0.009	0.015	0.027
JH (m)	0.005	0.008	0.015	-	-	-	0.005	0.008	0.014	0.010	0.015	0.028	0.005	0.009	0.016	0.005	0.008	0.015
*Kinetic parameters*																		
PF (N∙kg^−1^)	-	-	-	0.26	0.40	0.73	0.30	0.47	0.84	-	-	-	-	-	-	-	-	-
MF (N∙kg^−1^)	-	-	-	0.16	0.26	0.47	0.10	0.16	0.47	-	-	-	-	-	-	-	-	-
mREFD (N∙s^−1^∙kg^−1^)	-	-	-	9.42	1.88	26.11	4.88	0.97	13.53	-	-	-	-	-	-	-	-	-
mRCFD (N∙s^−1^∙kg^−1^)	-	-	-	2.74	0.54	7.59	3.46	0.69	9.61	-	-	-	-	-	-	-	-	-
F@0V (N∙kg^−1^)	-	-	-	0.30	0.47	0.84	0.42	0.65	1.17	-	-	-	-	-	-	-	-	-
PP (W∙kg^−1^)	-	-	-	0.63	0.98	1.76	1.13	1.76	3.15	1.31	2.03	3.63	0.69	1.07	1.92	0.49	0.76	1.36
MP (W∙kg^−1^)	-	-	-	0.32	0.51	0.91	0.67	1.03	1.86	-	-	-	-	-	-	-	-	-
mRPD (W∙s^−1^)	-	-	-	11.38	2.27	31.54	0.30	0.06	0.84	-	-	-	-	-	-	-	-	-
MEccConP (W∙kg^−1^∙s^−1^)	-	-	-	0.29	0.45	0.80	0.25	0.40	0.71	-	-	-	-	-	-	-	-	-
TI (N∙s^−1^)	-	-	-	4.06	6.29	11.26	6.24	9.67	17.30	-	-	-	-	-	-	-	-	-
RNI (N∙s^−1^∙kg^−1^)	-	-	-	0.02	0.03	0.05	0.05	0.08	0.14	-	-	-	-	-	-	-	-	-

MCAP, motion capture system; ConD, concentric duration; CT, total contraction time; EccD, eccentric duration; F@0V, force at zero velocity; FT, flight time; FT:CT, flight time to contraction time ratio; MCD, minimal detectable change; MF, mean force; MP, mean power; MEccConP, mean eccentric and concentric power over time; MEF, mean eccentric force; MinV, minimum velocity; mRCFD, maximum rate of concentric force development; mRCED, maximum rate of eccentric force development; mRPD, maximum rate of power development; PF, peak force; PP, peak power; PV, peak velocity; RNI, relative net impulse; SEM, standard error of measurement; SWC, smallest worthwhile change; T2PF, time to peak force; T2PP, time to peak power; TI, total impulse; V@PP, velocity at peak power.
